# Rare Mould Fungaemia at a Tertiary Academic Hospital in Athens, Greece: A 15-Year Survey and Literature Review

**DOI:** 10.3390/jof11090644

**Published:** 2025-09-01

**Authors:** Maria Siopi, Angeliki Alevra, Dimitrios Mitsopoulos, Spyros Pournaras, Joseph Meletiadis

**Affiliations:** 1 Clinical Microbiology Laboratory, “Attikon” University General Hospital, Medical School, National and Kapodistrian University of Athens, 12462 Athens, Greece; marizasiopi@hotmail.com (M.S.); spournaras@med.uoa.gr (S.P.); 2 Molecular Microbiology and Immunology Laboratory, Department of Biomedical Sciences, University of West Attica, 12243 Athens, Greece; angelikealeura@gmail.com (A.A.); dimitris.mits01@gmail.com (D.M.)

**Keywords:** fungaemia, rare moulds, epidemiology, Greece

## Abstract

Invasive infections caused by rare moulds (RM) are increasingly reported and often exhibit resistance to antifungal agents. Their epidemiology varies regionally, yet data from Greece are scarce. To address this gap, we conducted a 15-year retrospective study of RM fungaemia at a tertiary academic hospital in Athens, Greece. All microbiologically confirmed cases in hospitalised patients between 2010 and 2024 were reviewed. Demographic and clinical data were retrieved from medical records. Incidence rates were calculated per 1000 admissions and 10,000 bed-days. Isolates were morphologically identified and, when available, molecularly characterised and tested for antifungal susceptibility according to EUCAST guidelines. Eight RM fungaemia episodes (0.8% of total fungaemias) were identified, with an incidence of 0.01/1000 admissions and 0.03/10,000 bed-days, without bacterial co-infections. Haematological malignancies (62%) were the most common underlying condition. *Fusarium* spp. were the predominant pathogens (6/8), followed by single cases due to *Lomentospora prolificans* and *Acremonium* spp. Amphotericin B showed the highest in vitro activity against *Fusarium* isolates (MIC 0.5–1 mg/L), followed by voriconazole (MICs 2–8 mg/L) whereas other azoles showed no in vitro activity (MICs ≥ 8 mg/L). Half of the infections were breakthrough, whereas in 3/8 cases, the diagnosis was established post-mortem (n = 2) or post-discharge. Among the five patients who received treatment, the crude mortality rate was 60%. This first epidemiological report on RM fungaemia in Greece highlights the predominance of *Fusarium* spp., the frequency of breakthrough infections, and the challenges in early diagnosis and management. Increased clinical awareness and regional surveillance are essential for optimising outcomes.

## 1. Introduction

In recent years, invasive infections caused by rare fungal pathogens have come under the spotlight, primarily owing to their association with high mortality rates [1,2]. Rare mould (RM) fungaemias are typically defined as those attributed to non-*Aspergillus* spp. and non-Mucorales when filamentous fungi are involved [3]. Of note, their epidemiology is influenced by multiple predisposing factors and exhibits marked geographical heterogeneity, possibly reflecting variations in clinical practices, access to diagnostic tools, and patient population characteristics [3,4]. Their laboratory diagnosis poses considerable challenges, including the need for experienced personnel, low sensitivity and prolonged turnaround time of blood cultures, limited availability of surrogate biomarkers, and restricted implementation of modern molecular or proteomic identification techniques, often due to financial constraints [3,4]. Moreover, the intrinsic resistance or reduced susceptibility of RM to currently licenced antifungal agents further complicates therapeutic management and has been linked to a notable incidence of breakthrough infections [5]. Meanwhile, the interpretation of antifungal susceptibility testing (AFST) results remains problematic, as clinical breakpoints have not been established for these pathogens [4,5].

Given these limitations, epidemiological investigation of RM fungaemia is critical in the era of widespread prophylactic and empirical antifungal use. Nevertheless, current data in the international literature are limited, often outdated, and predominantly derived from case reports, single-centre experiences, or small outbreak investigations [3]. At the national level, the epidemiological landscape in Greece remains poorly defined.

Based on these grounds, we performed a systematic review of the existing literature related to bloodstream infections attributed to RM in Greece and investigated their epidemiology in a tertiary academic hospital over the last 15 years to provide an overview of their burden, microbiological profiles, and clinical characteristics, thereby addressing the existing knowledge gap in the Greek epidemiological context.

## 2. Materials and Methods

### 2.1. Literature Review

Electronic literature searches were conducted in PubMed, Google Scholar, and Web of Science in July 2025, using the keywords ‘*Acremonium*’, ‘*Aureobasidium*’, ‘*Bisifusarium*’, ‘bloodstream’, ‘dematiaceous’, ‘disseminated’, ‘*Exophiala*’, ‘fungaemia’, ‘fusariosis’, ‘*Fusarium*’, ‘hyalohyphomycosis’, ‘*Lomentospora*’, ‘lomentosporiosis’, ‘*Neocosmospora*’, ‘*Paecilomyces*’, ‘*Penicillium*’, ‘phaeohyphomycosis’, ‘*Pseudallescheria*’, ‘*Purpureocillium*’, ‘*Rasamsonia*’, ‘scedosporiosis’, ‘*Scedosporium*’; ‘*Schizophyllum*’, ‘*Scopulariopsis*’, ‘*Talaromyces*’ and ‘*Wangiella*’ in combination with ‘Greece’ and/or ‘Greek’. Additionally, the reference lists of retrieved articles were screened for further relevant studies. Only articles published in English were included, with no restrictions on publication date. From each eligible study, the following data were extracted (when available): study setting and time frame, patient demographic characteristics (age and sex) and clinical background (underlying conditions and immune status), causative pathogens and antifungal susceptibility profile, antifungal treatment administered, and outcome.

### 2.2. Study Setting

A retrospective surveillance study of patients with RM fungaemia was conducted at “Attikon” University General Hospital, covering the period from 2010 to 2024. “Attikon” is a modern 750-bed tertiary care teaching hospital located in Attica, the most densely populated region of Greece, which encompasses the metropolitan area of Athens. The hospital includes adult, paediatric and neonatal intensive care units, haematology and oncology wards, as well as specialised units for bone marrow transplantation and HIV/AIDS care.

RM fungaemia was defined as the recovery of moulds other than *Aspergillus* spp. or Mucorales [3] from at least one blood culture obtained during hospitalisation. Demographic data (age and sex), hospital unit at the time of infection onset, underlying disease, mycological findings and patients’ outcomes were obtained from the hospital’s computerised databases. Information regarding antifungal therapy was extracted from paper-based medical charts. Neutropenia was defined as an absolute neutrophil count of <500 cells/μL sustained for ≥10 consecutive days. Prior antifungal exposure was defined as receipt of antifungal therapy for ≥7 days when there was first clinical suspicion of fungaemia.

### 2.3. Identification and AFST

Recovered isolates were initially identified at the genus and, when possible, species level based on their colonial characteristics (including texture, pigmentation of the obverse and reverse sides, and growth rate) and microscopic morphology. For stored strains (in sterile saline with 10% glycerol at −70 °C), sequence-based species identification was retrospectively carried out. Genomic DNA was extracted from fresh subcultures grown on Sabouraud glucose agar supplemented with gentamicin and chloramphenicol (Oxoid, Athens, Greece) using a column-based method (QIAamp^®^ DNA Mini Kit; Qiagen, Athens, Greece) that combined enzymatic (incubation with proteinase K at 56 °C for 10 min) and mechanical (vortexing with glass beads for 10 min) pre-treatment. The internal transcribed spacer (ITS) region ITS1-5.8S-ITS2 was amplified for all isolates, along with a portion of the β-tubulin gene for *Scedosporium* isolates and the translation elongation factor-1α (TEF-1α) gene for *Fusarium* isolates, using polymerase chain reaction (PCR) under previously described conditions [6], followed by sequencing.

AFST was conducted in accordance with the European committee for antimicrobial susceptibility testing (EUCAST) broth microdilution (BMD) reference methodology (E.Def 9.4) [7]. The antifungal agents tested included amphotericin B (Sigma-Aldrich, Athens, Greece), voriconazole (Pfizer Ltd., Kent, UK), posaconazole (Sigma-Aldrich, Athens, Greece), itraconazole (Sigma-Aldrich, Athens, Greece), isavuconazole (Sigma-Aldrich, Athens, Greece), anidulafungin (Pfizer, Groton, CT, USA), micafungin (Astellas Pharma, Tokyo, Japan) and caspofungin (Merck & Co., Rahway, NJ, USA). Final concentrations ranged from 0.008 to 8 mg/L for azoles and echinocandins, and 0.06 to 8 mg/L for amphotericin B. The recommended *Candida krusei* ATCC 6258, *C. parapsilosis* ATCC 22019, *Aspergillus flavus* ATCC 204304 and *A. fumigatus* ATCC 204305 were included as quality control strains. Microtitration plates were incubated at 35 ± 2 °C for 48 h (with an additional 24 h incubation required for *Scedosporium* spp. to obtain sufficient growth in the growth control). The minimum inhibitory concentration (MIC) endpoint for amphotericin B and azoles was defined as the lowest drug concentration resulting in complete visual inhibition of fungal growth compared to the drug-free control. For echinocandins, the minimum effective concentration was determined as the lowest drug concentration inducing morphological alterations of the hyphae compared to the growth control.

### 2.4. Statistical Analysis

The annual incidence of RM bloodstream infections was calculated by dividing the number of RM fungaemia episodes by the total number of fungaemia episodes recorded each year. Additionally, their incidence was expressed as the number of RM fungaemia episodes per 1000 hospital admissions and per 10,000 hospital bed-days, based on data obtained from the hospital’s administrative database. Descriptive statistics included medians and interquartile ranges (IQR) for continuous variables and absolute frequencies with corresponding percentages for categorical variables.

## 3. Results

### 3.1. Literature Review

We identified 9 published articles reporting a total of 22 cases of RM fungaemia in Greece, spanning the period from 1994 to 2024 (Table 1). All cases originated from single-centre studies conducted in public tertiary care hospitals. In particular, 5 studies described individual cases: 1 in a premature extremely low birth weight neonate [8], 1 in a paediatric haematology patient [9], 1 in a paediatric oncology patient [10], 1 in an adult haematology patient [11] and 1 in an adult with no known predisposing factors for invasive fungal infection [12]. One study reported a small case series (n = 2) in paediatric oncology patients that appeared to be epidemiologically related [13], while another presented surveillance data on fungal bloodstream infections (n = 5) in the general hospital population [14]. The remaining two studies detailed outbreak investigations, one among 3 haematopoietic stem cell transplant recipients [15] and the other involving 7 non-neutropenic adults [16].

(i) **Patients’ characteristics**. Demographic and clinical data were available for 17 of the 22 patients (77%). Among these, 76% (13/17) were male, with a median (range, IQR) age of 60 (0.5–86, 70) years. Haematologic malignancies were reported in 35% (6/17). Most patients had venous catheters (94%, 16/17), either central (n = 12) or peripheral (n = 4), and were receiving antibiotic therapy (88%, 15/17). Concomitant Gram-negative bacteraemia was documented in 24% (4/17). Invasive mould infections typically affect hosts with compromised immune systems, as observed in the majority of patients (53%, 9/17). Notably, however, 8 patients were non-neutropenic or immunocompetent. Among these, 6 cases constituted a cluster following hospital renovation (all elderly individuals aged 63–86 years, admitted with urosepsis, endocarditis, pneumonia or bacteraemia) [16]; 1 case occurred in a critically ill premature neonate [8] and another in an adult without identifiable risk factors for invasive fungal infection [12] (Table 1).
(ii) **Species**. The causative pathogens belonged to the genera *Fusarium* (59%, 13/22), *Acremonium* (27%, 6/22), *Exophiala* (9%, 2/22) and *Scedosporium* (5%, 1/22). Among the *Fusarium* isolates, 7 were identified as *F. verticillioides*, 5 as *Fusarium* spp., and 1 as *F. musae*. The *Acremonium* isolates included 3 *A. kiliense* and 3 *Acremonium* spp., while 2 isolates were identified as *E. dermatitidis*, and 1 as *S. boydii*. Identification methods were available for 17/22 isolates and comprised conventional phenotypic techniques (colony morphology and microscopy) in 18% (3/17), and molecular amplification with sequencing of the ITS region or the TEF-1α gene (for *Fusarium* spp.) in 82% (14/17) (Table 1).
(iii) **Antifungal susceptibility.** AFST was performed on 13 isolates: 7 *F. verticillioides*, 3 *A. kiliense*, 2 *E. dermatitidis* and 1 *F. musae*. Standardised CLSI BMD testing of *F. verticillioides* isolates showed no in vitro activity for the tested antifungals, with MICs > 16 mg/L for all three echinocandins, amphotericin B, 5-flucytosine, itraconazole and posaconazole, and >1 mg/L for voriconazole. For *A. kiliense*, gradient diffusion strips indicated that voriconazole was the only agent with in vitro activity (MIC 0.5 mg/L). For *E. dermatitidis*, AFST using both EUCAST BMD and gradient diffusion strips methods showed that azoles (0.06–0.5 mg/L), except fluconazole (8–16 mg/L), and amphotericin B (0.25 mg/L) were active in vitro. The *F. musae* isolate, tested using the EUCAST BMD method, exhibited high MICs for amphotericin B, 5-flucytosine and all three echinocandins, moderate MICs for itraconazole (4 mg/L) and isavuconazole (1 mg/L), and low MICs for posaconazole and voriconazole (0.5 mg/L each) (Table 1).
(iv) **Antifungal therapy.** Breakthrough infections were documented in 35% (6/17) of cases, including 3 patients who had received one of the following regimens: conventional amphotericin B (1 mg/kg/d for 21 days), liposomal amphotericin B (3 mg/kg every 48 h for >5 days, then 5 mg/kg/d for an unspecified number of days) and fluconazole (3 mg/kg/d twice weekly for 40 days). Treatment details were not available for the remaining 3 patients. Voriconazole monotherapy was administered to 59% of patients (10/17), including 6 with *F. verticillioides*, 3 with *A. kiliense* and 1 with *F. musae*. Among them, 5 (50%) died: 3 with *F. verticillioides*, 1 with *A. kiliense* and 1 with *F. musae*. Additionally, one patient with *F. verticillioides* who received no antifungal therapy, one with *S. boydii* treated with conventional amphotericin B (100 mg twice daily for 25 days) and one with *E. dermatitidis* who received liposomal amphotericin B (7 mg/kg/d) in combination with fluconazole (6 mg/kg every 48 h) for 8 days, also succumbed. In contrast, the *E. dermatitidis*-infected patient who was treated sequentially with caspofungin (50 mg/d for an unspecified number of days) followed by voriconazole (8 mg/kg/d for an unspecified number of days, then 9 mg/kg orally twice daily for 14 days), survived. Another patient with *Fusarium* spp. survived after sequential therapy with conventional amphotericin B (1 mg/kg/d for 5 days) and caspofungin (50 mg/d for 21 days). Two *Acremonium* spp.-infected patients received liposomal amphotericin B (1 mg/kg/d for 2–4 days, then 5 mg/kg/d for 26–28 days) followed by fluconazole (5 mg/kg/d orally for 30 days) and survived. The overall crude mortality rate was 47% (8/17) (Table 1).

### 3.2. Single-Centre Experience from “Attikon” University General Hospital

Over a 15-year period (2010–2024), a total of 670,875 hospital admissions were recorded at “Attikon” (mean annual admissions: 44,725 patients). During this time, 990 episodes of fungaemia were documented, the vast majority of which were attributed to *Candida* spp. (95%, 944/990).

(i) **Incidence**. Eight episodes were caused by RM, corresponding to an overall incidence rate of 0.8% (range: 0–3.4%). The estimated incidence density was 0.01 (range: 0–0.04) episodes per 1000 hospital admissions and 0.03 (range: 0–0.10) episodes per 10,000 patient-days. All RM bloodstream infections occurred in patients admitted to internal medicine wards and were sporadically distributed throughout the study period (1 case each in 2011, 2012, 2015, 2016, 2019, and 2020, and 2 cases in 2017 spanning nine months), with no evidence of temporal or spatial clustering (Table 2).
(ii) **Patient characteristics.** Regarding patient demographics, 75% (6/8) were male, with a median (range, IQR) age of 64 (27–83, 25) years. Haematological malignancies were the most common underlying condition (5/8, 62%). At the time of diagnosis, all patients were febrile, and all but one (88%) were either neutropenic or immunosuppressed. In addition, all patients were receiving antibiotic therapy and had venous catheters (7 central and 1 peripheral). No bacterial bloodstream co-infections were recorded (Table 2).
(iii) **Biomarkers**. The median (range, IQR) C-reactive protein (CRP) level at the time of RM-positive blood culture sampling, available for all patients, was 113 (15–256, 167) mg/L, while the corresponding procalcitonin level, available for 4/8 patients, was 0.55 (0.40–6.46, 2) ng/mL. 1,3-β-D-glucan (BDG) testing (Fungitell^®^ assay, Associates of Cape Cod, East Falmouth, MA, USA) was performed in two patients within ±1 day of RM-positive blood culture collection, yielding one positive (692 pg/mL; breakthrough *N. keratoplastica* infection, fatal outcome) and one negative (48 pg/mL; *B. dimerum* infection, patient survived) result; no follow-up BDG measurements were conducted. The median (range, IQR) time from hospital admission to the diagnosis of RM fungaemia was 29 (4–88, 25) days.
(iv) **Species**. Overall, the median (range, IQR) time to blood culture positivity was 5 (3–10, 2) days. The predominant etiological agents were *Fusarium* spp. (including *Neocosmospora* spp. according to the updated nomenclature; 75%, 6/8), comprising 2 isolates each of *Bisifusarium dimerum* (formerly *F. dimerum*), *N. keratoplastica* (formerly *F. keratoplasticum*), and *F. oxysporum*. Single isolates of *Lomentospora prolificans* and *Acremonium* spp. were also identified. Mixed fungaemia with *N. keratoplastica* and *C. parapsilosis* occurred in one case, while all other episodes involved a single RM species. Molecular identification and AFST were successfully performed for all isolates except the *Acremonium* spp., which had not been stored (Table 2).
(v) **Antifungal susceptibility.** Among *Fusarium* isolates, amphotericin B was the only antifungal agent demonstrating consistent good in vitro activity (MIC 0.5–1 mg/L). Taking into account the EUCAST epidemiological cut-off value of amphotericin B for the *Neocosmospora* genus (formerly *F. solani* species complex; 8 mg/L), to which *N. keratoplastica* belongs, the *N. keratoplastica* isolates fall within the wild-type population. Voriconazole MICs were elevated, ranging from 2 to 8 mg/L, with *N. keratoplastica* isolates exhibiting the highest MIC of 8 mg/L, whereas other azoles showed no in vitro activity, with MICs ≥ 8 mg/L. In contrast, none of the antifungal agents tested were active in vitro against *L. prolificans* (Table 2).
(vi) **Antifungal therapy**. Half of the patients (4/8) were receiving antifungal therapy at the time of fungaemia onset, including 2 on mould-active prophylaxis. Antifungal treatment for the management of fungaemia was not administered in 3 cases, either due to death prior to blood culture results (n = 2) or discharge before diagnosis, in which case the infection outcome remains unknown. Of the 5 patients who received antifungal therapy, voriconazole monotherapy was used in 2 cases (40%), 1 with *L. prolificans* (no available information on drug’s dose) and 1 with *F. oxysporum* (4 mg/kg twice daily for 17 days), both of whom died. One patient infected with *N. keratoplastica* received a short-course salvage combination of liposomal amphotericin B (5 mg/kg/d) and isavuconazole (200 mg three times a day), which was discontinued after two days due to clinical deterioration and death. On the contrary, a patient infected with *B. dimerum* survived following 35 days of liposomal amphotericin B monotherapy (5 mg/kg/d). Another patient with *F. oxysporum* also survived after a sequential therapy with liposomal amphotericin B (5 mg/kg/d for 5 days), followed by combination therapy with the same dose of liposomal amphotericin and voriconazole (4 mg/kg twice a day) for 18 days (Table 2).
(vii) **Mortality**. The median (range, IQR) length of hospital stay following collection of the RM-positive blood culture was 18 (3–62, 29) days, whereas among patients who succumbed, the median (range, IQR) length of hospital stay from culture collection to death was 4 (3–62, 25) days. The overall crude mortality was 71% (5/7 patients with known outcomes), but when considering only those who received antifungal therapy, the corresponding rate decreased to 60% (3/5) (Table 2).

## 4. Discussion

RM fungaemia, although uncommon, constitutes a significant clinical concern. This study provides a comprehensive overview of the infection over a 15-year period within a general hospital setting. RM fungaemias accounted for a small yet clinically relevant proportion of all fungaemia episodes (0.8%), occurring sporadically without any clear outbreak pattern. *Fusarium* spp. predominated as the causative agents, primarily affecting neutropenic or immunocompromised patients, particularly those with underlying haematological malignancies. Half of the cases represented breakthrough infections, while diagnosis was established post-mortem or post-discharge in 38% of cases. Overall crude mortality was high at 71%, decreasing to 60% among patients who received antifungal treatment after diagnosis.

Recent literature and international guidelines have raised concerns about the escalating burden of invasive diseases caused by RM [3,4], likely driven by evolving host profiles, novel forms of immunosuppression and the selective pressure exerted by the extensive use of broad-spectrum, mould-active antifungal prophylaxis [17,18]. Indeed, the World Health Organization has categorised *Fusarium* spp. as high-priority fungal pathogens associated with systemic infections, while *Scedosporium* spp. and *L. prolificans* are listed among medium-priority threats [19]. However, the epidemiology of RM infections varies substantially both between and within countries, influenced by differences in patient populations, diagnostic capabilities and antifungal management practices [3,4], rendering regional epidemiological monitoring essential [20].

Accurately estimating the incidence of RM fungaemia remains challenging due to the paucity of long-term surveillance data. Our literature review showed that, in Greece, available data are limited to isolated case reports and two documented outbreaks over a 30-year period (1994–2024), precluding any reliable estimation of national incidence. To our knowledge, this is the first attempt to report the incidence of the infection in the general hospital population in Greece. We found that RM accounted for 0.8% of all positive fungal blood cultures at our institution over a 15-year period, a rate that is slightly higher than those reported in general patient populations (0.1–0.5%) [21,22,23], but lower than those observed in high-risk groups, such as patients with haematological malignancies or solid organ cancers (1.4–6.0%) [24,25,26,27].

Despite its limited occurrence, RM fungaemia carry notable clinical significance, particularly among immunocompromised individuals. This is underscored by the predominance of cases, both in our cohort and in the Greek literature reviewed, among patients with a history of haematological malignancy, which is consistent with global data emphasising the vulnerability of this population to opportunistic mould infections [5,28], largely due to prolonged neutropenia and intensive immunosuppressive therapies. However, our review also identified cases in non-neutropenic or immunocompetent individuals, particularly in the context of a hospital renovation-related outbreak [16], highlighting the role of environmental factors in the pathogenesis of the disease. This observation aligns with existing evidence that environmental disturbances, such as construction or renovation activities, can increase airborne fungal spore concentrations and facilitate nosocomial transmission [29]. *Fusarium* has been found in water, faucets, and on shower and sink surfaces, as well as in indoor air, serving as potential reservoirs for in-hospital outbreaks. Nosocomial outbreaks caused by *L. prolificans* appear to be uncommon and have been linked solely to the airborne transmission of the fungus [30]. In light of this, there is a clear need for robust environmental surveillance and infection control measures in healthcare facilities undergoing structural work. Furthermore, clinicians should maintain a high index of suspicion for invasive mould infections even in patients without traditional predisposing conditions when such environmental risk factors are present.

RM fungaemia presents formidable diagnostic challenges, owing to the low index of suspicion, the rarity of causative pathogens, and the limited sensitivity and slow turnaround of conventional diagnostic methods. Prompt diagnosis is paramount, as delays in pathogen identification can significantly impact therapeutic decisions and patient outcomes, especially in settings where empirical antifungal coverage may not target resistant or uncommon moulds such as *Fusarium* or *Lomentospora*. In our cohort, the median time to blood culture positivity was 5 days, with delayed growth (>5 days) observed in 25% of cases, consistent with previous studies indicating slower growth kinetics for these moulds compared to common yeasts like *Candida* spp. [28,31,32]. This observation further supports the consideration of extending blood culture incubation to up to 10 days in selected high-risk patients, particularly when no alternative microbiological documentation is available [28].

Of note, diagnosis was established post-mortem or after discharge in 38% of cases, reflecting significant diagnostic delays. The absence of specific fungal biomarkers [3,5], combined with the often non-specific clinical presentation, especially in patients without classical risk factors, further complicates early recognition. The rapid turnaround and non-invasive nature of common inflammatory proteins render them valuable tools for the early diagnosis of bloodstream infections. The median CRP levels among all patients in our cohort (113 mg/L; no bacterial co-infection) align with those previously reported in febrile patients with confirmed fungaemia (mean 113 ± 69 mg/L) [33], thereby supporting its potential role as an inflammatory marker in this setting. In contrast, the median procalcitonin level in our cohort (0.55 ng/mL; available for 4/8 patients) was half of that observed in the same patient population (median 1.15 ng/mL) [33]. However, low procalcitonin (<0.5 ng/mL) combined with elevated CRP (100–300 mg/L) has been shown to serve as a potential biomarker for invasive fungal infections in immunocompromised patients with haematological malignancies, similar to our cohort, offering optimal specificity, sensitivity, and positive and negative predictive values (81%, 85%, 73%, and 89%, respectively) [34].

As for established serological fungal biomarkers, their diagnostic value in RM fungaemia remains marginal. In particular, serum galactomannan may yield positive results in invasive fusariosis (sensitivity 7–83%) [35,36]. Nevertheless, due to potential cross-reactivity with *Aspergillus*, particularly in high-incidence settings, results should be interpreted with caution when distinguishing between these infections. Galactomannan testing is not recommended for *L. prolificans* due to the absence of the antigen in its cell wall. Regarding BDG, a recent meta-analysis showed that it may aid in the detection of fungaemia attributed to *Fusarium* spp. (sensitivity 81%) and *Scedosporium* spp./*L. prolificans* (sensitivity 75%), albeit this should be interpreted with caution, considering local epidemiology and pre-test probability. Meanwhile, its specificity and positive predictive value remain key concerns, especially in non-hematologic patients or when used for screening in high-risk haematologic populations, and thus BDG is considered more useful for ruling out rather than confirming the infections [37]. In our cohort, BDG testing was performed in 2/8 patients within 24 h of RM-positive blood culture sampling. One patient with *N. keratoplastica* infection had a markedly elevated BDG level (692 pg/mL), whereas the other, infected with *B. dimerum*, tested negative (48 pg/mL). Although available data are limited, no species-specific BDG patterns have been described [38].

Incorporating rapid molecular diagnostics, such as species-specific PCR, could substantially reduce time to diagnosis and enable earlier initiation of targeted therapy. Serum PCR for *Fusarium* DNA has been shown to have high specificity (100%) and sensitivity (93%) for diagnosing invasive haematogenous fusariosis, enabling detection a median of 6 days earlier than blood culture or biopsy [35]. The sensitivity of serum PCR for detecting *L. prolificans* DNA has been reported at 83% [39]. This is of critical importance given the high mortality associated with invasive RM infections and the frequent resistance of these pathogens to first-line antifungals, which often renders empirical therapy inadequate. Nevertheless, it should be acknowledged that molecular-based diagnostic testing for RM is not widely available in routine clinical practice and is currently performed mainly as in-house testing at specialised centres with mycology expertise [3,5].

In line with previous reports, *Fusarium* spp. and related genera accounted for the vast majority of RM fungaemias in our cohort (6/8 episodes) [22,28]. Although primarily plant pathogens, these ubiquitous environmental fungi are well-recognised opportunists in immunocompromised hosts. Fusariosis is distinctive among mould infections for its frequent bloodstream involvement, reflecting a strong propensity for vascular invasion. Species within the *Neocosmospora* genus, especially *N. falciformis* and *N. keratoplastica*, are responsible for ~50% of invasive fusariosis cases, while the *F. oxysporum* species complex accounts for ~20% [40]. Reflecting this distribution, 67% (4/6) of *Fusarium*-related infections in our cohort were caused by these species. A case of *L. prolificans*-related fungaemia was also identified, consistent with the organism’s known propensity for haematogenous dissemination. *L. prolificans* is an environmental mould typically found in hot and semi-arid regions, including southern Europe, and is increasingly recognised as a cause of invasive fungal disease, particularly in immunocompromised individuals [40].

The antifungal susceptibility profile of RM pathogens is marked by intrinsic multidrug resistance, complicating therapeutic choices and often leaving limited effective options. To date, available MIC data remain scarce and the absence of established clinical breakpoints further hampers interpretation [4,5]. In our cohort, AFST revealed that amphotericin B was the only agent with consistent good in vitro activity against *Fusarium* isolates (MIC 0.5–1 mg/L). Voriconazole displayed elevated and species-dependent MICs (MIC 2–8 mg/L), with the highest value observed in *N. keratoplastica* (8 mg/L), while other azoles and echinocandins were uniformly ineffective (MIC ≥ 8 mg/L). These findings align with global data indicating that amphotericin B tends to have lower MICs compared to azoles [40], and that voriconazole susceptibility varies across *Fusarium* spp., particularly among *Neocosmospora* spp. that frequently demonstrate higher resistance [40], highlighting the importance of species-level identification to inform antifungal therapy [5]. Nevertheless, the clinical utility of AFST in invasive fusariosis remains controversial, as correlation between MIC and treatment outcome is inconsistent, with persistent neutropenia and corticosteroid use identified as strong predictors of mortality [41]. Notably, *L. prolificans*, which was also identified in our cohort, demonstrated pan-resistance to all tested agents, consistent with its well-documented multidrug-resistant profile and poor responsiveness to conventional antifungals [42]. As such, AFST for both fusariosis and lomentosporiosis is currently strongly recommended for epidemiological surveillance and marginally advised for MIC-guided therapy [3].

Given the aforementioned details, RMs exhibit high levels of resistance to most antifungal classes, rendering breakthrough infections difficult to predict and manage. Mould-active prophylaxis, while intended to reduce the risk of invasive fungal disease, may inadvertently select for intrinsically resistant organisms, thereby contributing to breakthrough events. In our cohort, breakthrough fungaemia occurred in 4/8 patients, in line with previously reported trends [28], including 2 who were receiving mould-active prophylaxis (voriconazole or isavuconazole) at the time of onset. Notably, prior exposure to voriconazole has been identified as a significant independent predictor of breakthrough *Fusarium* fungaemia [43], while cases of isavuconazole breakthrough fusariosis have also been reported [44]. These findings bear significant implications in local settings such as Greece, where mould-active prophylaxis is extensively used (95%) among haematology patients [45], highlighting the need for ongoing surveillance, rigorous antifungal stewardship, and the development of centre-specific management strategies informed by the interpretation of local expertise and epidemiological evidence.

International guidelines strongly recommend voriconazole or liposomal amphotericin B as first-line therapy for fusariosis and voriconazole-based combination antifungal therapy (particularly with terbinafine) as first-line treatment for infections caused by *L. prolificans* [3]. In our cohort, voriconazole monotherapy was used in two fatal cases involving *L. prolificans* and *F. oxysporum*. A patient with *N. keratoplastica* received a short two-day course of liposomal amphotericin B combined with isavuconazole, but passed away during treatment. In contrast, 2 patients infected with *B. dimerum* and *F. oxysporum* survived following treatment with liposomal amphotericin B alone or in sequential combination with voriconazole, respectively. Despite antifungal treatment, crude mortality among our patients remained high at 60% (50% for *Fusarium* spp./*Neocosmospora* spp. and 100% for *L. prolificans*), mirroring global estimates ranging from 43% to 100% [28,40,42], with clinical outcomes primarily determined by host immune status [42,46].

The present study has several limitations that should be acknowledged. First, it was conducted in a single tertiary care centre, which may limit the generalizability of the findings to other institutions or healthcare settings, both within Greece and internationally. Although our hospital serves a large and diverse patient population, regional variations in epidemiology and antifungal management practices may exist [3,4]. In addition, the retrospective design hampered the availability of certain clinical data, particularly regarding environmental exposures, host-related factors, extent of organ involvement and infection-attributable mortality. Furthermore, the small number of episodes precluded robust statistical analyses of risk factors and outcomes. While this reflects the rarity of the infection, it limits the ability to draw definitive conclusions about optimal treatment approaches or prognostic indicators.

Nonetheless, this study represents the first attempt to assess the epidemiology of RM fungaemia within the general hospital population in Greece, providing valuable baseline data and shedding light on a previously undocumented clinical burden. As such, our findings may serve as a critical reference point for future surveillance efforts.

## 5. Conclusions

This first epidemiological report on RM fungaemia in Greece highlights the predominance of *Fusarium* spp., the notable incidence of breakthrough infections, and the obstacles in prompt diagnosis and treatment, underscoring the need for heightened clinical awareness, particularly among at-risk populations. Future multicentre surveillance studies, both locally and globally, are warranted to further elucidate epidemiology, treatment responses and prognostic factors. The high mortality rates emphasise the pressing need for novel antifungal development and enhanced therapeutic strategies targeting these uncommon yet formidable pathogens.

## Figures and Tables

**Table 1:** Reported cases of fungaemia caused by rare moulds in Greece, presented in chronological order.

No	Year of Diagnosis (City)	Sex/Age (Years)	Underlying Disease and Risk Factors (Immune Status)	Causative Agent (Identification Method)	Antifungal Susceptibility (Testing Method)	Breakthrough Infection (Previous Antifungal, Duration)	Antifungal Therapy	Outcome	Reference
1	1994 (Thessaloniki)	M/2	Neuroblastoma, CTX, CVC, ABT (neutropenic)	*Acremonium* spp. (colonial/microscopic morphology)	NA	No	LAMB (30 days; 1 mg/kg/d × 4 days, then 5 mg/kg/d) → FLC (30 days; 5 mg/kg/d po)	Survival (defervescence by day 6 of LAMB, negative cultures by day 10 of LAMB)	[13]
2	1994 (Thessaloniki)	F/4	ALL, CVC, ABT (neutropenic)	*Acremonium* spp. (colonial/microscopic morphology)	NA	No	LAMB (30 days; 1 mg/kg/d × 2 days, then 5 mg/kg/d) → FLC (30 days; 5 mg/kg/d po)	Survival (defervescence by day 4 of LAMB, negative cultures by day 8 of LAMB)	[13]
3	2003 (Thessaloniki)	M/67	AML, CTX, CVC, ABT (neutropenic)	*Fusarium* spp. (colonial/microscopic morphology)	NA	Yes (AMB, 1 mg/kg/d × 21 days)	AMB (1 mg/kg/d × 5 days) → CAS (50 mg/d × 21 days)	Survival (defervescence and negative cultures by day 7 of CAS)	[11]
4	2009 (Thessaloniki)	F/60	Possible acute myocardial infarction, 1-month Russian spa treatment 1 month prior (non-neutropenic)	*Scedosporium boydii* (ITS sequencing)	NA	No	AMB (100 mg bid × 25 days)	Death due to cardiorespiratory arrest	[12]
5	2009–2018 (Athens)	NA	NA	*Acremonium* spp. (NA)	NA	NA	NA	NA	[14]
6	2009–2018 (Athens)	NA	NA	*Fusarium* spp. (NA)	NA	NA	NA	NA	[14]
7	2009–2018 (Athens)	NA	NA	*Fusarium* spp. (NA)	NA	NA	NA	NA	[14]
8	2009–2018 (Athens)	NA	NA	*Fusarium* spp. (NA)	NA	NA	NA	NA	[14]
9	2009–2018 (Athens)	NA	NA	*Fusarium* spp. (NA)	NA	NA	NA	NA	[14]
10	2011 (Thessaloniki)	F/39	Allogeneic HSCT recipient, acute GVHD, CVC, ABT (immunosuppressed)	*Acremonium kiliense* (ITS sequencing)	FLC > 256 mg/L, AFG/ITC/PSC/CAS/5-FC > 32 mg/L, AMB 32 mg/L, VRC 0.5 mg/L (gradient diffusion strips)	Yes (NA)	VRC (NA)	Survival	[15]
11	2011 (Thessaloniki)	F/33	Autologous HSCT recipient, relapsed Hodgkin’s disease, CVC, ABT (immunosuppressed)	*Acremonium kiliense* (ITS sequencing)	FLC > 256 mg/L, AFG/ITC/PSC/CAS/5-FC > 32 mg/L, AMB 32 mg/L, VRC 0.5 mg/L (gradient diffusion strips)	Yes (NA)	VRC (NA)	Survival	[15]
12	2011 (Thessaloniki)	M/23	Allogeneic HSCT recipient, Gram-negative pneumonia, CVC, ABT (immunosuppressed)	*Acremonium kiliense* (ITS sequencing)	FLC > 256 mg/L, AFG/ITC/PSC/CAS/5-FC > 32 mg/L, AMB 32 mg/L, VRC 0.5 mg/L (gradient diffusion strips)	Yes (NA)	VRC (NA)	Death due to underlying condition	[15]
13	2012 (Larissa)	M/74	Gram-negative UTI/BSI, diabetes mellitus, PVC, ABT (non-neutropenic)	*Fusarium verticillioides* (ITS and TEF-1α sequencing)	AMB/ITC/5-FC > 32 mg/L, PSC 32 mg/L, AFG/CAS/MFG > 16 mg/L, VRC 1 mg/L (CLSI BMD)	No	VRC (4 mg/kg bid × 24 days)	Death	[16]
14	2012 (Larissa)	M/85	Gram-negative UTI/BSI, CVC, ABT (non-neutropenic)	*Fusarium verticillioides* (ITS and TEF-1α sequencing)	AMB/ITC/5-FC > 32 mg/L, PSC 32 mg/L, AFG/CAS/MFG > 16 mg/L, VRC 1 mg/L (CLSI BMD)	No	VRC (NA)	Survival	[16]
15	2012 (Larissa)	M/86	Endocarditis, CVC, ABT (non-neutropenic)	*Fusarium verticillioides* (ITS and TEF-1α sequencing)	AMB/ITC/5-FC > 32 mg/L, PSC 32 mg/L, AFG/CAS/MFG > 16 mg/L, VRC 1 mg/L (CLSI BMD)	No	No	Death	[16]
16	2012 (Larissa)	M/70	ITP, PVC, corticosteroid therapy (immunosuppressed)	*Fusarium verticillioides* (ITS and TEF-1α sequencing)	AMB/ITC/5-FC > 32 mg/L, PSC 32 mg/L, AFG/CAS/MFG > 16 mg/L, VRC 1 mg/L (CLSI BMD)	No	VRC (NA)	Death	[16]
17	2012 (Larissa)	M/63	Gram-negative pneumonia, PVC, ABT (non-neutropenic)	*Fusarium verticillioides* (ITS and TEF-1α sequencing)	AMB/ITC/5-FC > 32 mg/L, PSC 32 mg/L, AFG/CAS/MFG > 16 mg/L, VRC 1 mg/L (CLSI BMD)	No	VRC (NA)	Death	[16]
18	2012 (Larissa)	M/82	Gram-negative pneumonia, PVC, ABT (non-neutropenic)	*Fusarium verticillioides* (ITS and TEF-1α sequencing)	AMB/ITC/5-FC > 32 mg/L, PSC 32 mg/L, AFG/CAS/MFG > 16 mg/L, VRC 1 mg/L (CLSI BMD)	No	VRC (NA)	Survival	[16]
19	2012 (Larissa)	M/80	Gram-negative BSI, CVC, ABT (non-neutropenic)	*Fusarium verticillioides* (ITS and TEF-1α sequencing)	AMB/ITC/5-FC > 32 mg/L, PSC 32 mg/L, AFG/CAS/MFG > 16 mg/L, VRC 1 mg/L (CLSI BMD)	No	VRC (NA)	Survival	[16]
20	2021 (Athens)	M/0.5	ELBW, Gram-negative BSI, abdominal surgery, PN, CVC, ABT, (non-neutropenic)	*Exophiala dermatitidis* (ITS sequencing)	FLC 16 mg/L, AFG/MFG 4 mg/L, CAS 2 mg/L, ISA 0.5 mg/L, AMB 0.25 mg/L, VRC/ITC 0.125 mg/L, PSC 0.06 mg/L (EUCAST BMD)	Yes (FLC, 3 mg/kg/d biw × 40 days)	LAMB (7 mg/kg/d) + FLC (6 mg/kg q48h) × 8 days	Death	[8]
21	2022 (Crete)	M/4.5	Ewing’s sarcoma, 1 month post-CTX, CVC, ABT (immunosuppressed)	*Exophiala dermatitidis* (ITS sequencing)	AFG/MFG/CAS/5-FC > 32 mg/L, FLC 8 mg/L, AMB 0.25 mg/L, VRC 0.06 mg/L (gradient diffusion strips)	No	CAS (NA; 50 mg/d) → VRC (NA; 8 mg/kg/d × NA days, then 9 mg/kg bid po × 14 days)	Survival	[10]
22	2024 (Athens)	M/1.2	Refractory KMT2A-rearranged infant B-ALL, CTX, CVC, ABT (neutropenic)	*Fusarium musae* (ITS sequencing)	AMB/5-FC 32 mg/L, ITC 4 mg/L, ISA 1 mg/L, PSC/VRC 0.5 mg/L, AFG/CAS/MFG 8 mg/L (EUCAST BMD; ISA by gradient diffusion strips)	Yes (LAMB 3 mg/kg q48h × >5 days, then 5 mg/kg/d × NA days)	VRC (18 weeks; increased from 4 mg/kg bid to 15 mg/kg bid based on TDM)	Gradual resolution of symptoms/signs shortly after dose increase to 15 mg/kg bid—Death due to underlying disease	[9]

Abbreviations. ABT, antibiotic treatment; AFG, anidulafungin; ALL, acute lymphoblastic leukaemia; AMB, amphotericin B; AML, acute myeloid leukaemia; bid, twice daily; biw, twice weekly; BMD, broth microdilution; BSI, bloodstream infection; CAS, caspofungin; CLSI, clinical and laboratory standards institute; CTX, chemotherapy; CVC, central venous catheter; ELBW, extremely low birth weight; EUCAST, European committee on antimicrobial susceptibility testing; F, female; FLC, fluconazole; HSCT, haematopoietic stem cell transplant; ISA, isavuconazole; ITC, itraconazole; ITP, idiopathic thrombocytopenic purpura; ITS, internal transcribed spacer; LAMB, liposomal amphotericin B; M, male; MFG, micafungin; NA, not available; po, orally; PN, parenteral nutrition; PSC, posaconazole; PVC, peripheral venous catheter; q48h, every 48 h; TEF-1α, translation elongation factor-1α; TDM, therapeutic drug monitoring; UTI, urinary tract infection; VRC, voriconazole; 5-FC, 5-flucytosine.

**Table 2:** Reported cases of fungaemia caused by rare moulds in “Attikon” University General Hospital (2010–2024), listed in chronological order.

No	Year of Diagnosis	Sex/Age (Years)	Underlying Disease and Risk Factors (Immune Status)	Causative Agent (Identification Method)	Antifungal Susceptibility Based on EUCAST BMD	Breakthrough Infection (Previous Antifungal, Duration)	Antifungal Therapy	Outcome
1	2011	M/27	HSCT recipient, invasive rhinosinusitis, CVC, ABT (immunosuppressed)	*Lomentospora prolificans* (ITS and β-tubulin sequencing)	AFG/CAS/MFG > 8 mg/L, PSC/VRC/ITC/ISA > 8 mg/L, AMB 4 mg/L	Yes (FLC 400 mg/d × 25 days)	VRC (NA)	Death
2	2012	M/73	AML, CTX, CVC, ABT (neutropenic)	*Fusarium oxysporum* (ITS and TEF-1α sequencing)	AFG/CAS/MFG/ITC > 8 mg/L, ISA 8 mg/L, PSC/VRC 2 mg/L, AMB 0.5 mg/L	Yes (VRC 4 mg/kg bid × 10 days)	VRC (4 mg/kg bid × 17 days)	Death
3	2015	M/83	Decreased level of consciousness, aspiration pneumonia, PVC, ABT (non-neutropenic)	*Acremonium* spp. (colonial/microscopic morphology)	NA	No	No	Death *
4	2016	M/58	NHL, corticosteroid therapy, CVC, ABT (neutropenic)	*Neocosmospora keratoplastica* (ITS and TEF-1α sequencing)	AFG/CAS/MFG > 8 mg/L, PSC/ITC/ISA > 8 mg/L, VRC 8 mg/L, AMB 1 mg/L	No	No	Death *
5	2017	F/75	Pulmonary malignancy with cerebral metastases, CTX, CVC, ABT (neutropenic)	*Bisifusarium dimerum* (ITS and TEF-1α sequencing)	AFG/CAS/MFG > 8 mg/L, PSC/ITC/ISA > 8 mg/L, VRC 8 mg/L, AMB 1 mg/L	No	No	NA ^#^
6	2017	F/42	SLE, tuberculosis, corticosteroid therapy, CVC, ABT (immunosuppressed)	*Bisifusarium dimerum* (ITS and TEF-1α sequencing)	AFG/CAS/MFG > 8 mg/L, PSC/ITC/ISA > 8 mg/L, VRC 4 mg/L, AMB 0.5 mg/L	No	LAMB (5 mg/kg/d × 35 days)	Survival
7	2019	M/70	NHL, corticosteroid therapy, CVC, ABT (neutropenic)	*Neocosmospora keratoplastica* (ITS and TEF-1α sequencing) ^^^	AFG/CAS/MFG > 8 mg/L, PSC/ITC/ISA > 8 mg/L, VRC 8 mg/L, AMB 1 mg/L	Yes (AFG 100 mg/d × 3 days → CAS 50 mg/d × 7 days → LAMB 5 mg/kg/d × 4 days)	LAMB (5 mg/kg/d) + ISA (200 mg tid) × 2 days	Death
8	2020	M/51	AML, corticosteroid therapy, CVC, ABT (neutropenic)	*Fusarium oxysporum* (ITS and TEF-1α sequencing)	AFG/CAS/MFG/ITC/ISA > 8 mg/L, PSC/VRC 4 mg/L, AMB 1 mg/L	Yes (ISA 200 mg/d × 12 days)	LAMB (5 mg/kg/d × 5 days) → LAMB (5 mg/kg/d) + VRC (4 mg/kg bid) × 18 days	Survival

* Patient died before notification of positive blood culture. ^#^ Patient discharged before receiving positive blood culture results. ^^^ Mixed fungaemia with *Candida parapsilosis*. Abbreviations. AFG, anidulafungin; AMB, amphotericin B; AML, acute myeloid leukaemia; bid, twice daily; BMD, broth microdilution; CAS, caspofungin; EUCAST, European committee on antimicrobial susceptibility testing; F, female; FLC, fluconazole; HSCT, haematopoietic stem cell transplant; ISA, isavuconazole; ITC, itraconazole; ITS, internal transcribed spacer; LAMB, liposomal amphotericin B; M, male; MFG, micafungin; NA, not available; NHL, non-Hodgkin lymphoma; PSC, posaconazole; SLE, systemic lupus erythematosus; TEF-1α, translation elongation factor-1α; tid; three times a day; VRC, voriconazole.

## Data Availability

Data available on request. Accession numbers for sequences deposited in GenBank include OR400646, OR400706, OR400707, OR400739, OR400740, OR400759, and OR400739.
